# Is simultaneous placement of orthodontic mini-implants and skeletally anchored appliances advisable? CBCT-based cadaver study comparing the accuracy of the digital workflow with methods requiring two visits

**DOI:** 10.1007/s00056-025-00578-x

**Published:** 2025-03-04

**Authors:** Till Augustinowitz, Lucia Schiavon, Katharina Mücke, Beryl Schwarz-Herzke, Dieter Drescher, Giulia Brunello, Kathrin Becker

**Affiliations:** 1 Department of Orthodontics, University Hospital Düsseldorf, Moorenstraße 5, 40225 Düsseldorf, Germany; 2 Clinic of Reconstructive Dentistry, Center for Dental Medicine, University Zurich, Plattenstrasse 11, 8032 Zurich, Switzerland; 3 Department of Neurosciences, Dentistry Section, University of Padua, Via Giustiniani 2, 35128 Padua, Italy; 4 Department of Orthodontics and Dentofacial Orthopedics, Charité – Universitätsmedizin Berlin, corporate member of Freie Universität Berlin and Humboldt-Universität zu Berlin, Aßmannshauser Straße 4–6, 14197 Berlin, Germany; 5 Institute of Anatomy II, Medical Faculty, Heinrich Heine University, Universitätsstraße 1, 40225 Düsseldorf, Germany; 6 Department of Oral Surgery, University Hospital Düsseldorf, Moorenstraße 5, 40225 Düsseldorf, Germany

**Keywords:** Orthodontic anchorage procedures, Digital workflow, Ex vivo study, Optical impression, Cone-beam computed tomography, Kieferorthopädische Verankerungsverfahren, Digitaler Workflow, Ex-vivo-Studie, Optischer Abdruck, Digitale Volumentomographie

## Abstract

**Purpose:**

For the fabrication of an orthodontic mini-implant (OMI)-borne appliance, the position of the inserted OMI can be detected by a silicone impression or an intraoral scan (IOS). In case of digital planning, it can be taken over from the planning and the appliance can be produced in advance. This study aimed to evaluate the accuracy of these three techniques and whether there is an association with the insertion angle.

**Methods:**

Two OMIs were digitally planned and placed in the anterior palate of 11 human cadavers with different insertion angles. Subsequently, the position of each OMI was detected by an IOS, a silicone impression, and a cone-beam computed tomography (CBCT) scan, whereby the CBCT scan was set as “real position”. The measurements of accuracy were performed between the CBCT data as a reference and the preoperative digital planning, the IOS and the plaster model manufactured from the silicone impression.

**Results:**

The IOS was the most accurate in detecting the Top (mean deviation 0.14 mm) and the Apex (mean deviation 0.36 mm) of the OMIs. Significant linear deviations between the three modalities were registered for both Top (*p* < 0.001) and Apex (*p* = 0.010). The digital planning procedure achieved the lowest mean angular deviation of 3.7° and was significantly more accurate in this respect than the IOS (*p* < 0.001).

**Conclusion:**

All methods were subject to small, but clinically irrelevant deviations. Within the limitations of a cadaver study, all methods appear to be suitable for clinical use. However, the digital workflow could be advantageous, requiring only a single visit for OMI placement and simultaneous appliance fitting.

**Supplementary Information:**

The online version of this article (10.1007/s00056-025-00578-x) contains supplementary material, which is available to authorized users.

## Abstract (de)

**Zielsetzung:**

Zur Herstellung einer auf Mini-Implantaten befestigten kieferorthopädischen Apparatur kann die Position der Mini-Implantate durch einen Silikonabdruck oder einen Intraoralscan erfasst werden. Bei einer digitalen Planung kann sie aus der Planung übernommen und die Apparatur anhand dieser Planung hergestellt werden. Ziel dieser Studie war es, die Genauigkeit dieser 3 Techniken zu vergleichen und festzustellen, ob es einen Zusammenhang mit dem Insertionswinkel gibt.

**Methoden:**

Zwei Mini-Implantate wurden digital geplant und in den vorderen Gaumen von 11 Humanpräparaten mit unterschiedlichen Insertionswinkeln eingesetzt. Anschließend wurde die Position jedes Mini-Implantats durch einen Intraoralscan, einen Silikonabdruck und eine DVT(digitale Volumentomographie)-Aufnahme ermittelt. Die Position in der DVT wurde als „reale Position“ festgelegt. Die Genauigkeitsmessungen wurden zwischen den DVT-Daten als Referenz und der digitalen Planung, dem Intraoralscan und dem aus dem Silikonabdruck hergestellten Gipsmodell durchgeführt.

**Ergebnisse:**

Der Intraoralscan erwies sich als am genauesten bei der Erkennung des Kopfes (mittlere Abweichung: 0,14 mm) und des Apex (mittlere Abweichung: 0,36 mm) der Mini-Implantate. Signifikante lineare Abweichungen zwischen den 3 Techniken wurden sowohl für den Kopf (*p* < 0,001) als auch für den Apex (*p* = 0,010) festgestellt. Die digitale Planung erzielte die geringste mittlere Angulationsabweichung von 3,7° und war diesbezüglich signifikant genauer als der Intraoralscan (*p* < 0,001).

**Schlussfolgerungen:**

Bei allen Methoden traten kleine, aber klinisch irrelevante Abweichungen auf. Mit den Einschränkungen einer Kadaverstudie scheinen alle Methoden für den klinischen Einsatz geeignet zu sein. Die komplett digitale Planung könnte jedoch vorteilhaft sein, da nur ein einziger Termin für das Inserieren der Mini-Implantate und das Einsetzen der Apparatur notwendig ist.

## Introduction

Orthodontic mini-implants (OMIs) have gained increasing popularity as a tool for skeletal anchorage of orthodontic appliances due to their ease of use, minimally invasive insertion, and the reduced need for patient compliance. Furthermore, they allow the insertion of a variety of appliances with good control of three-dimensional (3D) tooth movements [1, 2].

One of the most utilized sites for OMI insertion is the anterior palate, especially in the T‑zone located immediately posterior to the palatal rugae. Indeed, this zone is characterized by good bone thickness and a limited risk of interference with teeth and other relevant structures [3].

OMI-supported appliances are conventionally manufactured on a plaster model obtained from a silicone impression [4]. More recently, digital technologies have revolutionized the fabrication of these appliances. The position of the OMIs can be obtained with an intraoral scanner (IOS) and the appliances can be digitally designed and produced using additive manufacturing, such as laser melting printing. Afterwards, the orthodontic appliance is placed in a second appointment [5].

In addition, the OMI position can be digitally planned for guided insertion based on a 3D model of the upper jaw, as an alternative to freehand insertion, and it is possible to design the orthodontic appliance directly at the same time. This approach permits appliance placement immediately after OMI insertion in a single appointment [6, 7]. However, the fitting of this appliance largely depends on the accuracy of OMI placement.

Therefore, the primary aim of this study on human frozen maxillae was to investigate the accuracy of OMIs placed paramedian in the anterior palate using digitally planned tooth-borne guides and to compare it with the accuracy of OMI detection by means of a subsequent IOS or conventional silicone impression.

The secondary aim was to examine whether the insertion angle of the OMIs had an influence on the accuracy of the three methods mentioned above (i.e., IOS, silicone impression, and digital planning) since in implant dentistry the implant angle does not seem to affect the accuracy when IOS is utilized [8].

## Materials and methods

### Preparation of the human cadaver

Eleven frozen heads of human cadavers were utilized. To improve the working field at the anterior palate, the mandible, the tongue, and the calvaria up to the orbits were removed.

In cases of insufficient residual dentition, the maxillary dentition in the premolar and molar region was restored using a resin-based restoration fixed to the maxilla with lateral fixation pins (Art. No. 010.6124, Institut Straumann AG, Basel, Switzerland) to provide sufficient support for a tooth-borne OMI insertion guide. To this aim, a cone-beam computed tomography (CBCT) scan (Orthophos SL 3D, Dentsply Sirona, York, PA, USA) and an IOS (TRIOS 3, 3Shape, Copenhagen, Denmark) were taken. The resin base of the restoration was designed with coDiagnostiX® (DentalWings, Institut Straumann AG) and then 3D-printed. Subsequently, the dental arch was set up on the base using resin teeth. Representative images of a resin-based restoration are provided in Suppl. Fig. 1.

### Digital planning of the OMI position and production of the insertion guides

For each sample, two OMIs (2 × 9 mm, BENEfit® System, PSM Medical GmbH, Gunningen, Germany) were digitally planned on a maxillary IOS (TRIOS 3, 3Shape). In the samples that had received a resin-based restoration, a new IOS was taken after fixation of the prosthesis for OMI planning.

**Fig. 1:**
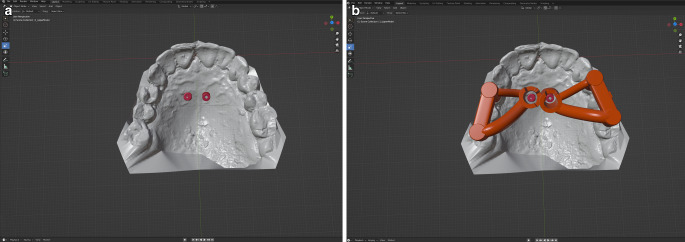
Digital planning: **a** the orthodontic mini-implants (*OMIs*) were planned with insertion angles between 0 and 30° to the occlusal plane; **b** design of an OMI insertion guide Digitale Planung: **a** Die kieferorthopädischen Mini-Implantate wurden mit Insertionswinkeln zwischen 0 und 30° zur Okklusionsebene geplant; **b** Design eines Insertions-Guides für kieferorthopädische Mini-Implantate

The OMIs were planned at insertion angles between 0 and 30° to the occlusal plane in the paramedian area of the anterior palate in the anterior part of the T‑zone using the software Blender (Blender Foundation, Amsterdam, The Netherlands; Fig. 1).

An insertion guide was designed and 3D printed in resin (Surgical Guide Resin, Formlabs Inc., Sommerville, MA, USA) using an SLA printer (Form 3, Formlabs Inc.). A total of 22 OMIs were planned, of which 21 were used for measurements, i.e., 3 OMIs for each insertion angle (0, 5, 10, 15, 20, 25, and 30°). Details of the procedures are provided in Suppl. Table 1. The distribution of the insertion angles was chosen so that two OMIs with the same insertion angle were not inserted in the same sample.

### Ex vivo insertion and postoperative capturing

**Fig. 2:**
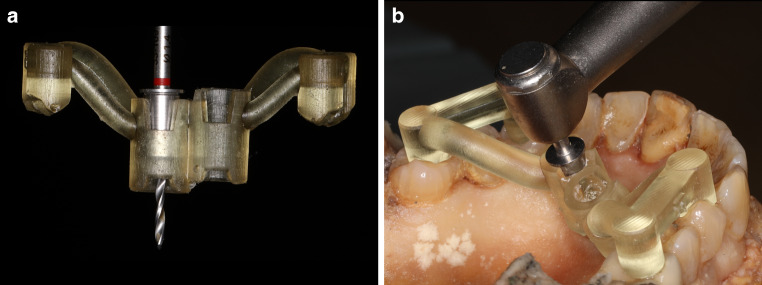
The pilot drill (**a**) and the insertion tool (**b**) were provided with a vertical stop, allowing the insertion of the orthodontic mini-implant at the desired depth Vorbohrer (**a**) und Insertionsinstrument (**b**) wurden mit einem vertikalen Anschlag versehen, der das Inserieren der kieferorthopädischen Mini-Implantate in der gewünschten Tiefe ermöglicht

Predrilling was performed with a pilot drill and the OMIs were inserted using a torque-limited handpiece (iSD900, NSK, Tochigi, Japan). The correct insertion depth was guaranteed by a vertical stop on the pilot drill and the insertion tool (Fig. 2). A postoperative CBCT was taken using the following acquisition parameters: 85 kV, 12 mA, exposure time 14.4 s, 8 × 8 cm FOV, voxel size 160 μm. The OMI position was recorded three times with an IOS (TRIOS3, 3Shape) without scan bodies. Afterwards, a biphasic silicone impression was taken (Express STD-Putty + Express 2 Light BodyFlow, 3M, St. Paul, MN, USA) with impression caps (BENEfit® System, PSM Medical GmbH). Hard plaster casts were obtained and digitized with a laboratory scanner (E4, 3Shape). A flowchart summarizing the study design is presented in Suppl. Fig. 2.

### Accuracy of OMI position detection with IOS and silicone impression

For each sample, the .dicom file from postoperative CBCT and .stl files of the three postoperative IOSs and of the digitized plaster model were imported into OnyxCeph (Image Instruments, Chemnitz, Germany). All measurements were performed by the same operator (T.A.) using a 23.8 inch, Full HD (1920 × 1080), 75 Hz monitor (Acer Vero RL242Y).

Each .stl file (IOSs and digitized plaster model) was aligned to the occlusal plane and matched to the CBCT to evaluate the accuracy of the IOS and silicone impression, as compared to the CBCT scan that was used as a reference to represent the real OMI position. For superimposition, the radiopaque structures of the jaw and the fixation pins of the resin-based restorations were used as reference points without considering the position of the OMIs.

**Fig. 3:**
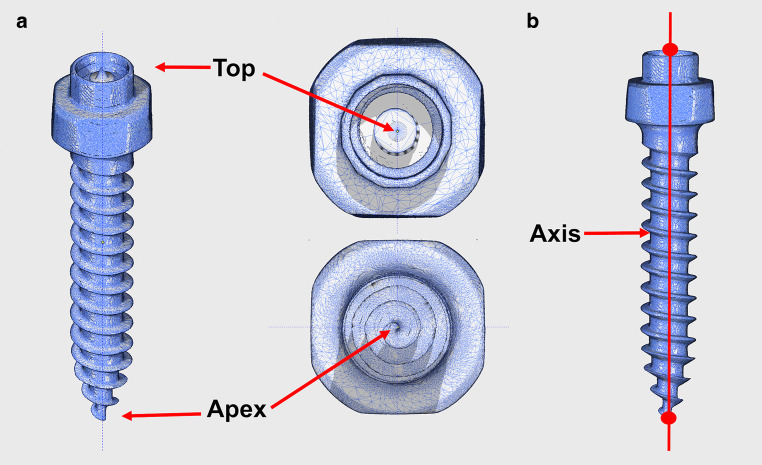
**a** Identification of the points *Top* and *Apex*. **b** Vertical axis of the orthodontic mini-implant passing through the points *Top* and *Apex* **a** Definition der Punkte *Top* und *Apex*. **b** Vertikale Achse der kieferorthopädischen Mini-Implantate, die durch die Punkte *Top* und *Apex* verläuft

For each CBCT/IOS and CBCT/digitized plaster model evaluation, an .stl file of the OMI provided by the manufacturer was matched to the OMIs and used for measurements. A cone was designed with Blender on the top of each OMI, with its axis corresponding to the vertical axis of the OMI. The vertex of the cone was identified as *Top*. Then, the most apical point of each OMI, along its vertical axis, was determined and referred to as *Apex*. These two points were used for measurements and allowed a line passing through the vertical axis of the OMI to be drawn (Fig. 3).

For each sample, all three IOSs and the silicone impression were measured separately. The right and left OMIs were also evaluated individually.

The respective distances at the *Top* and *Apex* were measured in mm, representing the discrepancies between the techniques. In addition, the position of the points *Top* and *Apex* was exported as a vector to assess the deviation in a coordinate reference system, allowing the identification of the direction of the deviation in the three axes (i.e., X: transversal; Y: vertical/insertion depth; Z: sagittal).

Furthermore, the deviation of the angulation of the OMI as compared to the CBCT was determined in degrees based on the vertical axis of the OMI defined by *Top* and *Apex*.

### Accuracy of digital planning

**Fig. 4:**
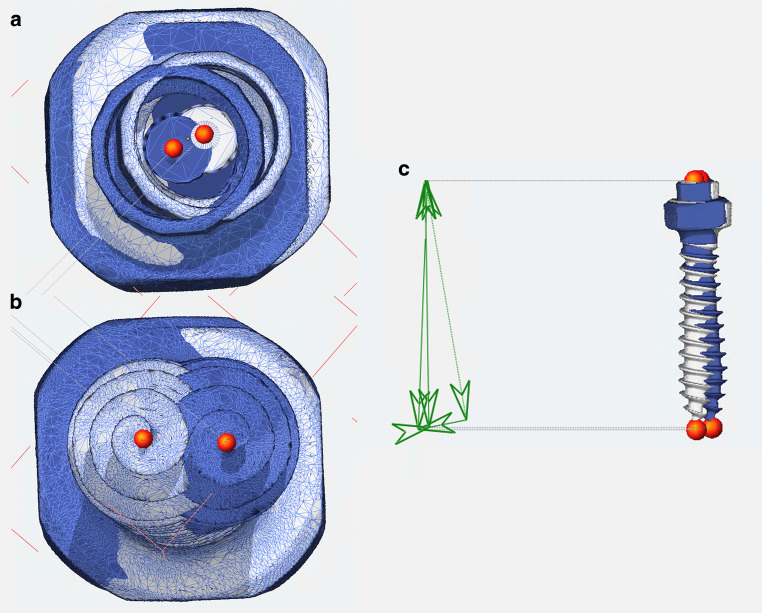
**a** Measurement of the deviation at the *Top*. **b** Measurement of the deviation at the *Apex*. **c** Measurement of the angular deviation **a** Messung der Abweichung am Punkt *Top*. **b** Messung der Abweichung am *Apex*. **c** Messung der Abweichung vom Insertionswinkel

The maxillary scans including the planned OMIs (see section “Digital planning of the OMI position and production of the insertion guides”) were exported from Blender, imported into OnyxCeph (Image Instruments), aligned to the occlusal plane, and matched with the postoperative CBCT. As previously described in the section “Accuracy of OMI position detection with IOS and silicone impression”, for both the planning and the CBCT an .stl of the OMI was aligned to the position of each OMI. The deviation between the planned position and that detected at the postoperative CBCT was computed for each OMI at the *Top* and at the *Apex* and in terms of angular deviation (Fig. 4).

### Statistical analysis

Statistical analysis was performed using R [9]. For intraexaminer calibration for each analyzed OMI, one randomly selected measurement on IOS data was repeated after at least 15 days using the icc-function from the irr-package (model = “two-way”, type = “consistency”). Boxplots were created to summarize the absolute values of the measured data, considering the pooled data from the repeated IOSs. Linear mixed effect models were used to analyze the effect of the three techniques (i.e., digital planning, silicone impression, or an IOS) and of the planned OMI angulation (0 to 30°) on linear (*Top* and *Apex*) and angular deviations. Additionally, the impact on linear deviations was assessed per x‑, y‑, and z‑direction. The absolute amount of the measured values was considered. Regarding linear deviation, data were pooled per axis (x-, y‑, z‑) and/or measurement position (*Apex*/*Top*) when needed. The technique and the OMI angulation were considered as fixed effects, whereas intercepts for the cadavers were defined as random effects representing the variability resulting from each donor. *P*-values were obtained by likelihood ratio tests (analysis of variance, ANOVA) of the full model with the effects in question, i.e., of the interaction between technique and angulation, as well as of technique separately. In case of significance, the Tukey post hoc test was carried out. Visual inspection of residual and Q–Q plots revealed no obvious deviations from homoscedasticity or normality. Results were considered significant at *p* < 0.05.

## Results

In 5 cases (samples no. 1, 2, 3, 4, and 5 of Suppl. Table 1), the manufacturing of resin-based restorations was required. All OMIs presented no mobility after insertion, including one OMI inserted accidentally in the nasopalatine canal, as revealed by the postoperative CBCT.

### Linear deviation—*Top* and *Apex*

Repeated linear measurements resulted in a very good intraclass correlation (icc = 0.886, *p* < 0.001), revealing high reliability. A summary of the linear deviations is provided in Suppl. Table 2.

Taking into consideration the overall linear deviation, pooling the data of deviations at the *Top* and *Apex*, IOS was found to be the most accurate method, followed by silicone impression and digital planning, with a mean linear deviation from the real position (postoperative CBCT) of 0.25, 0.38, and 0.52 mm, respectively.

**Fig. 5:**
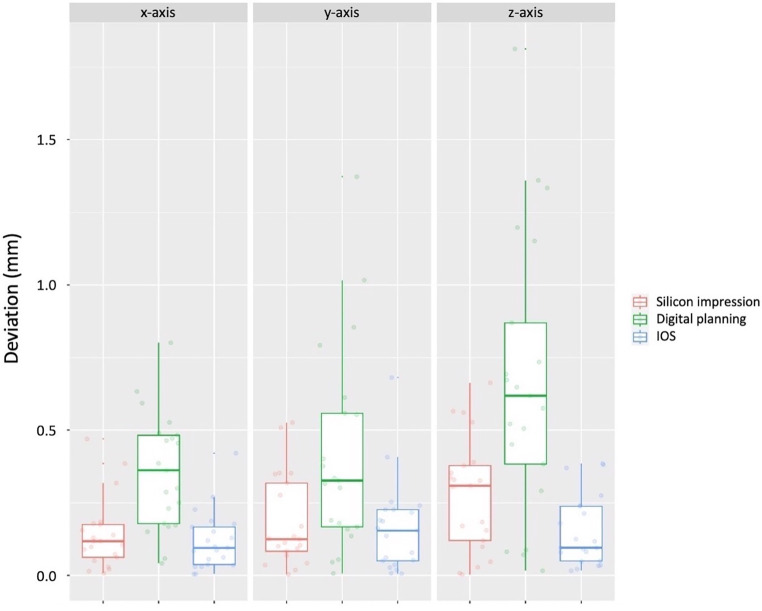
Measured deviation at the *Top* using digital planning, intraoral scan (*IOS*), and silicone impression Gemessene Abweichung am Punkt *Top* bei Verwendung der digitalen Planung, des Intraoralscans (*IOS*) und der Silikonabformung

The detection of the *Top* was most accurate using IOS, which presented a mean deviation from the real position of 0.14 mm, followed by silicone impression (0.20 mm) and digital planning (0.48 mm; Fig. 5). Statistical analysis confirmed significant differences between the modalities (*p* < 0.001).

**Fig. 6:**
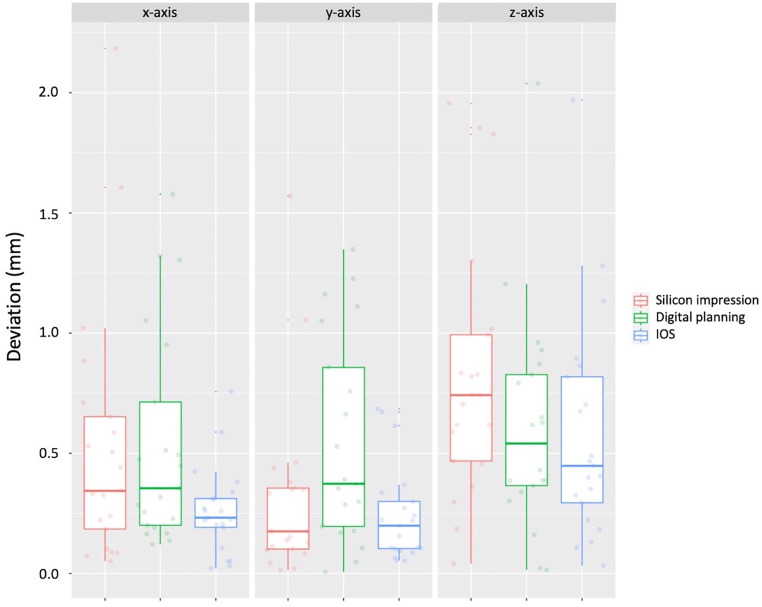
Measured deviation at the *Apex* using digital planning, intraoral scan (*IOS*), and silicone impression Gemessene Abweichung am *Apex* bei Verwendung der digitalen Planung, des Intraoralscans (*IOS*) und der Silikonabformung

The best results were also registered with IOS at the *Apex *with a mean total deviation of 0.36 mm (Fig. 6). Similar mean values at the *Apex* were recorded for digital planning (0.57 mm) and silicone impression (0.56 mm). A significant deviation at the *Apex* between the digital planning, IOS, and silicone impression was registered (*p* = 0.010).

The adjusted *p*-values for both the *Top* and *Apex* measurements are reported in Suppl. Table 3.

Regarding the direction of the deviation, at the *Top,* significant differences were found between the three techniques for all the tested axes (*p* ≤ 0.001; Suppl. Table 4), with digital planning exhibiting lower accuracy in all directions as compared to both IOS and silicone impression.

At the *Apex,* the differences between the groups were not so pronounced, with only one significant difference registered in the y‑direction between IOS and digital planning (*p* = 0.008). Despite the likelihood ratio test having also revealed significant differences in the x‑direction, these were not confirmed after performing the post hoc test.

### Angular deviation

**Fig. 7:**
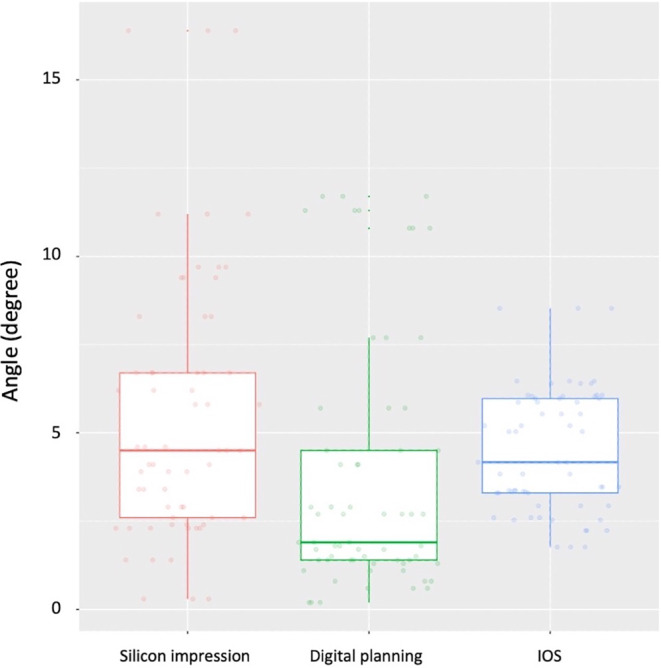
Measured angular deviation using digital planning, intraoral scan (*IOS*), and silicone impression Gemessene Winkelabweichung bei Verwendung der digitalen Planung, des Intraoralscans (*IOS*) und der Silikonabformung

As regards angular deviation (Suppl. Table 5), the lowest mean deviation was achieved using digital planning (3.71°). IOS reached a mean deviation of 4.51° and the silicone impression of 5.48° (Fig. 7). The latter also presented the highest maximum deviation value equal to 16.40°. Statistical analysis revealed a significant difference between the three entities (*p* < 0.001). The results of the post hoc tests are given in Suppl. Table 6.

### Influence of the insertion angle on linear and angular deviations

The interaction of the insertion angle and the use of different techniques (i.e., digital planning, IOS, silicone impression) had a significant impact on the overall linear accuracy (*p* < 0.001), as well as at the *Top* (*p* < 0.001), and *Apex* (*p* = 0.010), separately. Similarly, a significant difference was detected for angular deviation (*p* < 0.001). Post hoc tests confirmed significant differences only for an insertion angle of 15° for both linear and angular measurements (Suppl. Table 7), with lower deviations found in the IOS group for all parameters.

## Discussion

The primary aim of this study was to investigate the accuracy of OMI positioning using digitally planned tooth-borne insertion guides as well as the accuracy of IOS in comparison to a conventional silicone impression for detecting OMIs placed paramedian in the anterior palate. For producing an OMI-borne appliance, the detection of the *Top*, which provides the most relevant information, was most accurate using IOS, followed by silicone impression. However, digital planning also achieved a mean deviation of 0.48 mm at the *Top*, which can still be considered clinically acceptable based on clinical experience. However, it must be noted that evidence is lacking to what extent discrepancies between the planned and achieved positions can be tolerated.

The secondary aim was to examine whether the insertion angle of the OMIs has an influence on the accuracy. It could be shown that only the use of an insertion angle of 15° had a significant influence on the accuracy and that also, among the 15° measurements, IOS was the most accurate technique.

Inaccuracy of computer-guided implant surgery using tooth-supported drill guides based on preoperative IOS and CBCT scans has been reported in implant dentistry [10]. In order to transfer this knowledge to orthodontics, different aspects must be considered, such as the design and the material of the insertion guides. Möhlhenrich et al. found that the accuracy of the insertion guide can be increased by extension of the guide involving more teeth [11]. Furthermore, Mang de La Rosa et al. confirmed the suitability of 3D-printed insertion guides using the same resin utilized in the present study for direct placement of the orthodontic appliance after OMI insertion [12]. Basing the selection of our guide material and the tooth-borne design on the successful application in the above-mentioned studies, the low deviations at the *Top* obtained in all groups further support the potential immediate placement of the orthodontic appliance on the OMIs in a single appointment [13].

Additional inaccuracy may occur during guided insertion when too much pressure forces the insertion tool to have a false angle [14].

Another reason for an improper fit of a digitally planned orthodontic appliance is an excessive time interval between the preoperative IOS and the appointment of the fitting. The appliance and the insertion guide can deviate because of possible patients’ growth or variations in the position of the teeth [15].

An additional IOS or silicone impression would be necessary when digital planning has not been performed or in case of replacement of the orthodontic appliance utilizing the same OMIs as anchorage in the sense of multipurpose use [16].

In recent years, 3D technologies have gained popularity in orthodontics due to related improvements in patient care, the reduced planning time and the optimized workflow [17]. Performing an IOS results in a quicker workflow and brings advantages in terms of costs and time. It is probable that the IOS will become the option of choice for clinicians over traditional methods in the near future [18].

Therefore, this study aimed to investigate whether a conventional silicone impression could be replaced by an IOS. The present findings showed even a slightly higher accuracy with IOS at the OMI *Top* compared to the silicone impression. It must be taken into consideration that the performance of ex vivo scanning is easier for the operator than in clinical settings.

In addition, no scan bodies were used when performing the IOS in the present study. Clinical practice has shown that these can be dispensed with [19].

In general, if there is a minor discrepancy in the fit between the OMIs and the connector of the appliance, small chairside adjustments of the appliance connector are possible. These can be minimally widened by using a milling cutter and the rigid printed parts can be bent slightly [15].

Age correlates positively with a higher mucosa thickness and should not significantly influence the bone level in the selected paramedian region [20]. Nevertheless, the retention of the OMI might be reduced in situations with a thicker mucosa upon loading [21].

Indeed, one OMI was placed in the incisal canal, as revealed by the postoperative CBCT. The malpositioning of this OMI can be explained by the study design, which did not consider the ideal insertion angle [14].

The use of a lateral cephalogram combined with an IOS for planning the position of the OMIs in the anterior palate could be useful for the assessment of the vertical bone height [22, 23]. Since the anatomy of the anterior palate was found to exhibit a typical pattern, no CBCT is needed for digital OMI planning within the T‑zone in the majority of cases [14, 24]. In the present study, neither a lateral cephalogram nor a CBCT were used for planning the OMI positions. The postoperative CBCTs confirmed the efficiency of the procedure, since no damage to roots was observed, and OMIs were surrounded by a sufficient amount of bone tissue. A CBCT, however, might be indicated when OMIs are planned in palatal areas rather than in the T‑zone, in the presence of palatally displaced or retained teeth, or in the suspicion of inadequate vertical palatal bone height based on the routine cephalogram [22, 25]. In this respect, the development of low-dose CBCT protocols might allow more frequent use of 3D imaging to reducing the risk of injury to sensitive anatomical structures in the future [26].

A limitation of this study is represented by the age of the donors. Most of the human cadavers presented prosthetically reconstructed extended edentulous spaces or abraded teeth, which could not guarantee the same insertion guide stability as the teeth of common young orthodontic patients. Additionally, it has to be noted that the cadavers had been freshly frozen prior to the study and were defrosted to room temperature for the experiments. As the hardening of the silicone is temperature-dependent, the defrosting process might constitute a limitation. To mitigate the effect of the temperature, impressions were kept longer in the oral cavities than in clinical settings. Finally, a slight deviation may have derived from the superimposition of the IOS with the postoperative CBCT, as well as from the superimposition of the inserted OMIs with the .stl file of the OMI. Minimal metal artifacts were detected by the examiner deriving from a combination of partial volume effect, beam hardening, and Compton scatter. Nevertheless, they should have only negligibly contributed to deviations affecting the reliability of the CBCTs as references to validate the placement of the mini-implants.

## Conclusion

Within the limitations of this ex vivo study, all three applied methods appeared to be suitable for clinical use. Deviations, especially at the orthodontic mini-implant’s (OMI) *Top* which is the most important region for the fitting of an orthodontic appliance, were acceptably low with all methods. Despite the fact that the use of intraoral scan (IOS) after OMI insertion seemed to be the most accurate among the three investigated methods, the use of the other two approaches can also be recommended based on the current evidence. In daily practice, digital workflow could be advantageous over the other methods, since it would enable OMI placement and appliance fitting in a single appointment. Further investigations could evaluate the potential benefit of using scan bodies for IOS. Additionally, since the insertion angle had a significant influence only at 15°, no universally valid advice can be given in this regard.

## Supplementary Information

Supplementary figures 1–2 and Supplementary tables 1–7

## Data Availability

Data will be provided upon reasonable request.
