# Supplementary material for: Is simultaneous placement of orthodontic mini-implants and skeletally anchored appliances advisable? CBCT-based cadaver study comparing the accuracy of the digital workflow with methods requiring two visits
Source: J Orofac Orthop. 2025 Mar 4;87(3):269–78. doi: 10.1007/s00056-025-00578-x (PMC13109175; doi:10.1007/s00056-025-00578-x)
Supplement: Supplementary file 1 — Supplementary figures 1–2 and Supplementary tables 1–7 [file 56_2025_578_MOESM1_ESM.pdf]

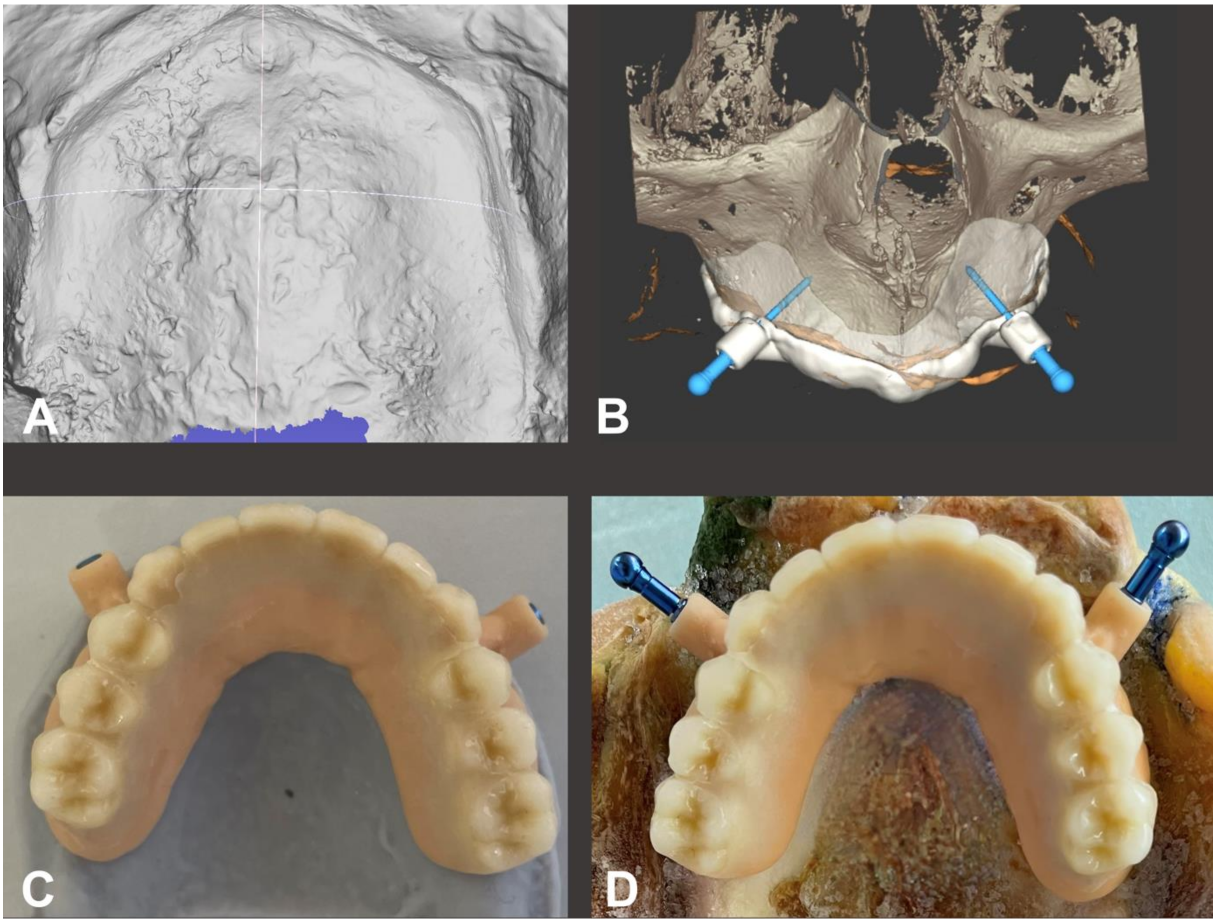

Suppl. Figure 1. Representative edentulous case receiving a resin-based restoration prior to the experiments. (A) Preoperative scanning. (B) Digital planning using IOS and CBCT. (C) Resin-based restoration on the model. (D) Resin-based restoration stabilized with fixation pins.

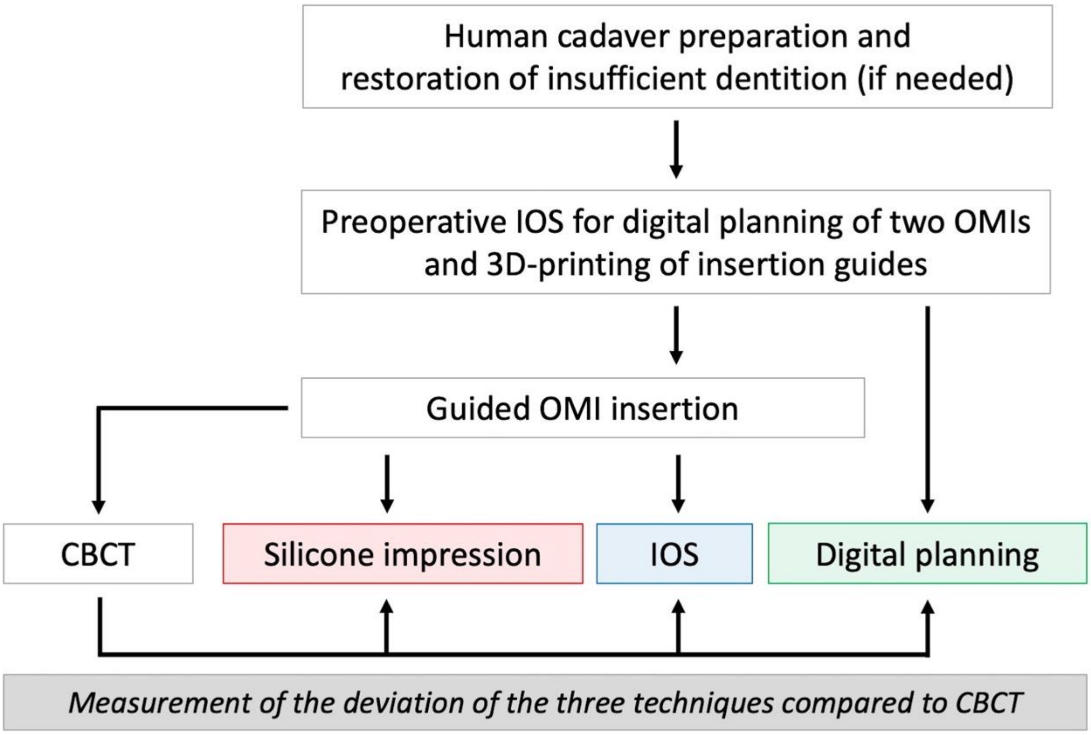

Suppl. Figure 2. Flowchart summarizing the study design.

Suppl. Table 1. OMI insertion angles including the randomization.

Sample	Insertion angle left OMI	Insertion angle right OMI
**1**	0°	5°
**2**	0°	5°
**3**	0°	5°
**4**	15°	25°
**5**	15°	25°
**6**	15°	25°
**7**	10°	20°
**8**	10°	20°
**9**	10°	30°
**10**	30°	20°
**11**	30°	25° (*)

* not analyzed

Suppl. Table 2. Overall linear deviations and deviations of the Top and Apex registered using digital planning, IOS and silicone impression. Mean, standard deviation (SD), minimum (min), maximum (max), median, and 95% confidence interval (95% CI) are reported.

Technique	N	Criterion	Mean	SD	Min	Max	Median	95% CI
**Digital planning**	21	**Overall linear measurements, i.e., *Top* and *Apex* (mm)**						
		All axes	0.52	0.41	0.01	2.04	0.40	0.20-0.71
		x	0.34	0.26	0.00	0.84	0.31	0.11-0.51
		y	0.45	0.36	0.05	1.36	0.35	0.18-0.72
		z	0.50	0.46	0.00	1.93	0.46	0.16-0.71
		***Top* (mm)**						
		All axes	0.48	0.38	0.01	1.81	0.40	0.18-0.63
		x	0.36	0.20	0.04	0.80	0.36	0.18-0.48
		y	0.42	0.35	0.01	1.37	0.33	0.17-0.56
		z	0.67	0.48	0.02	1.81	0.62	0.38-0.87
		***Apex* (mm)**						
		All axes	0.57	0.43	0.01	2.04	0.39	0.24-0.84
		x	0.54	0.44	0.12	1.58	0.35	0.20-0.71
		y	0.55	0.42	0.01	1.35	0.37	0.20-0.86
		z	0.61	0.45	0.01	2.04	0.54	0.37-0.83
**IOS**	63	**Overall linear measurements, i.e., *Top* and *Apex* (mm)**						
		All axes	0.25	0.28	0.00	1.97	0.19	0.08-0.31
		x	0.12	0.10	0.00	0.38	0.11	0.05-0.16
		y	0.17	0.17	0.00	0.65	0.13	0.06-0.20
		z	0.27	0.26	0.01	1.02	0.21	0.09-0.31
		***Top* (mm)**						
		All axes	0.14	0.13	0.00	0.68	0.10	0.05-0.21
		x	0.12	0.10	0.00	0.42	0.09	0.04-0.17
		y	0.16	0.16	0.01	0.68	0.15	0.05-0.23
		z	0.15	0.12	0.02	0.38	0.10	0.05-0.24
		***Apex* (mm)**						
		All axes	0.36	0.34	0.02	1.97	0.26	0.12-0.44
		x	0.26	0.18	0.02	0.76	0.23	0.19-0.31
		y	0.24	0.20	0.05	0.69	0.20	0.10-0.30
		z	0.58	0.46	0.03	1.97	0.45	0.29-0.82
**Silicone impression**	21	**Overall linear measurements, i.e., *Top* and *Apex* (mm)**						
		All axes	0.38	0.42	0.00	2.18	0.26	0.10-0.50
		x	0.26	0.29	0.03	1.25	0.17	0.07-031
		y	0.18	0.20	0.01	0.78	0.11	0.04-0.25
		z	0.36	0.23	0.01	0.77	0.38	0.16-0.51
		***Top* (mm)**						
		All axes	0.20	0.17	0.00	0.66	0.13	0.08-0.33
		x	0.14	0.12	0.01	0.47	0.12	0.06-0.17
		y	0.19	0.16	0.00	0.53	0.12	0.08-0.32
		z	0.28	0.20	0.00	0.66	0.31	0.12-0.38

		***Apex* (mm)**						
		All axes	0.56	0.52	0.01	2.18	0.38	0.16-0.74
		x	0.53	0.54	0.05	2.18	0.34	0.19-0.65
		y	0.31	0.37	0.01	1.57	0.18	0.10-0.36
		z	0.82	0.53	0.04	1.96	0.74	0.47-0.99

Suppl. Table 3. Linear deviation at the Top and Apex. A mixed linear model was performed, and the adjusted p-values derived from the post-hoc test are reported and labeled as follows: * p < 0.05, ** p < 0.01, and *** p < 0.001.

Technique – variable	Comparator	P-value (*Top*)	P-value (*Apex*)
Silicone impression	Digital planning	< 0.001***	0.990
Silicone impression	IOS	0.392	0.029*
Digital planning	IOS	< 0.001***	0.020*

Suppl. Table 4. Linear deviations in the three axes. A mixed linear model was performed, and the adjusted p-values derived from the post-hoc test are reported and labeled as follows: * p < 0.05, ** p < 0.01, and *** p < 0.001.

Criterion	Direction	P-value (ANOVA)	Technique - variable	Comparator	P-value (post-hoc)
TOP	x	< 0.001***	Silicone impression	Digital planning	< 0.001***
			Silicone impression	IOS	0.869
			Digital planning	IOS	< 0.001***
	y	0.001**	Silicone impression	Digital planning	0.008**
			Silicone impression	IOS	0.950
			Digital planning	IOS	0.003**
	z	< 0.001***	Silicone impression	Digital planning	< 0.001***
			Silicone impression	IOS	0.293
			Digital planning	IOS	< 0.001***
APEX	x	0.038*	Silicone impression	Digital planning	0.999
			Silicone impression	IOS	0.077
			Digital planning	IOS	0.071
	y	0.007**	Silicone impression	Digital planning	0.052
			Silicone impression	IOS	0.758
			Digital planning	IOS	0.008**
	z	0.202	Silicone impression	Digital planning	/
			Silicone impression	IOS	/
			Digital planning	IOS	/

Suppl. Table 5. Angular deviations were registered using digital planning, IOS, and silicone impression. Mean, standard deviation (SD), minimum (min), maximum (max), median, and 95% confidence interval (95% CI) are reported. Data are expressed in degree (°).

Technique	N	Mean	SD	Min	Max	Median	95% CI
**Digital planning**	21	3.71	3.56	0.20	11.70	1.90	1.40-4.50
**IOS**	63	4.51	1.73	1.77	8.53	4.17	3.30-5.97
**Silicone impression**	21	5.48	3.76	0.30	16.40	4.50	2.60-6.70

Suppl. Table 6. Angular deviation. A mixed linear model was performed, and the adjusted p-values derived from the post-hoc test are reported and labeled as follows: * p < 0.05, ** p < 0.01, and *** p < 0.001.

Technique - variable	Comparator	P-value
Silicone impression	Digital planning	< 0.001***
Silicone impression	IOS	0.072
Digital planning	IOS	0.167

Suppl. Table 7. Influence of the insertion angle of 15°. A mixed linear model was performed, and the adjusted p-values derived from the post-hoc test are reported and labeled as follows: * p < 0.05, ** p < 0.01, and *** p < 0.001.

Technique – variable	Comparator	P-values			
		**Overall linear measurements**	***Top***	***Apex***	**Angular**
Silicone impression	Digital planning	0.409	< 0.001***	0.573	< 0.001***
Silicone impression	IOS	0.002**	0.435	0.002**	< 0.001***
Digital planning	IOS	< 0.001***	< 0.001***	0.041*	0.406
